# Influence of Eggshell Powder on the Properties of Cement-Based Materials

**DOI:** 10.3390/ma17071705

**Published:** 2024-04-08

**Authors:** Gui-Yu Zhang, Seokhoon Oh, Yi Han, Li-Yi Meng, Runsheng Lin, Xiao-Yong Wang

**Affiliations:** 1 Department of Integrated Energy and Infra System, Kangwon National University, Chuncheon-si 24341, Republic of Korea; zhangguiyu@kangwon.ac.kr (G.-Y.Z.); gimul@kangwon.ac.kr (S.O.); hanyii@kangwon.ac.kr (Y.H.); mengliyi@kangwon.ac.kr (L.-Y.M.); 2 Yunnan Key Laboratory of Disaster Reduction in Civil Engineering, Faculty of Civil Engineering and Mechanics, Kunming University of Science and Technology, Kunming 650500, China; linrunsheng@kust.edu.cn; 3 Department of Architectural Engineering, Kangwon National University, Chuncheon-si 24341, Republic of Korea

**Keywords:** eggshell powder, concrete, microstructure, sustainability

## Abstract

Replacing cement with industrial by-products is an important way to achieve carbon neutrality in the cement industry. The purpose of this study is to evaluate the effect of eggshell powder on cement hydration properties, and to evaluate its feasibility as a substitute for cement. The substitution rates of eggshell powder are 0%, 7.5%, and 15%. Studying the heat of hydration and macroscopic properties can yield the following results. First: The cumulative heat of hydration based on each gram of cementitious material falls as the eggshell powder content rises. This is a result of the eggshell powder’s diluting action. However, the cumulative heat of hydration per gram of cement rises due to the nucleation effect of the eggshell powder. Second: The compressive strengths of ES0, ES7.5, and ES15 samples at 28 days of age are 54.8, 43.4, and 35.5 MPa, respectively. Eggshell powder has a greater negative impact on the compressive strength. The effect of eggshell powder on the speed and intensity of ultrasonic waves has a similar trend. Third: As the eggshell powder content increases, the resistivity gradually decreases. In addition, we also characterize the microscopic properties of the slurry with added eggshell powder. X-ray Diffraction (XRD) shows that, as the age increases from 1 day to 28 days, hemicaboaluminate transforms into monocaboaluminate. As the content of the eggshell powder increases, FTIR analysis finds a slight decrease in the content of CSH. Similarly, thermogravimetric (TG) results also show a decrease in the production of calcium hydroxide. Although the additional nucleation effect of eggshell powder promotes cement hydration and generates more portlandite, it cannot offset the loss of portlandite caused by the decrease in cement. Last: A numerical hydration model is presented for cement–eggshell powder binary blends. The parameters of the hydration model are determined based on hydration heat normalized by cement mass. Moreover, the hydration heat until 28 days is calculated using the proposed model. The strength development of all specimens and all test ages can be expressed as an exponential function of hydration heat.

## 1. Introduction

The cement industry is one of the pillar industries of civil engineering. As a basic building material, cement can meet various needs of infrastructure construction in modern society. But, we should see that the cement industry also has shortcomings that cannot be ignored, such as emitting a large amount of CO_2_, causing a greenhouse effect and thus threatening the sustainable development of the environment [1,2,3]. The CO_2_ emissions from the cement industry account for approximately 6–8% of the global total anthropogenic CO_2_ emissions [4,5,6,7]. In addition, for every 1 kg of clinker produced, an additional 1.7 kg of raw materials is required [8].

In order to reduce the carbon emissions of concrete, researchers have proposed many methods, such as carbonization curing and the use of mineral admixtures. Tam et al. [9] proposed that carbonation curing can realize the utilization of carbon dioxide, and the carbonization of recycled aggregate concrete can effectively improve the performance of concrete. Lin et al. [10] found that stone powder and calcium carbonate powder can improve the efficiency of carbonization curing. Under carbonization curing conditions, most of the crystallized hydration products can be carbonized. Chen and Gao [11] found that appropriate carbonization curing can improve the early strength of permeable concrete, and carbonization curing can improve the transition zone between aggregate and paste.

In addition, the use of mineral admixtures can reduce the amount of cement and improve the long-term performance of concrete [12]. Researchers have conducted extensive research on various admixtures. Wang [13] found that fly ash and slag can improve the resistance of concrete to seawater erosion and can also enhance sustainability. Han [14] analyzed the strength development and CO_2_ emissions of fly ash blended concrete, and proposed a CO_2_ reduction method for concrete containing fly ash. Kapeluszna [15] reported that mineral additives can improve late age strength and are suitable to produce low CO_2_ emission cement-based materials.

Global egg production is constantly increasing, and is expected to produce over 8 million tons of eggshell waste annually [16]. A large amount of eggshell waste is not utilized before its disposal. Hard eggshells account for about one tenth of the total weight of eggs [16]. The eggshell is mainly composed of calcium carbonate. Recently, the use of eggshells in building materials has attracted the attention of researchers. The use of crushed eggshells in cement concrete can not only solve the problem of landfill but can also reduce carbon dioxide emissions from the use of cement. In addition, some studies have shown that eggshell powder as a component of cement-based materials can have positive effects [16].

Although researchers have conducted a lot of research on low-carbon concrete mixed with supplementary cementitious materials (SCMs), we find that, so far, previous research has mainly focused on traditional SCMs. Although some research has been conducted on the mechanical properties of cement-based materials with added eggshell powder [17], further necessary research is required on this topic. Moreover, most previous works have focused on experimental works. Studies regarding a numerical model of cement–eggshell powder binary blends are very limited. To fill this gap, this study shows both an experimental study and a numerical model. This study conducts a series of macro- and micro-studies and presents a hydration model on eggshell powder concrete. Through these studies, researchers can obtain the systematic impact of eggshell powder on the mechanical properties, hydration heat properties, and chemical composition of concrete; clarify the mechanism and rules of the action of eggshell powder on concrete properties; and promote the engineering application of eggshell powder concrete.

## 2. Materials and Methods

### 2.1. Material Characterization

**Figure 1:**
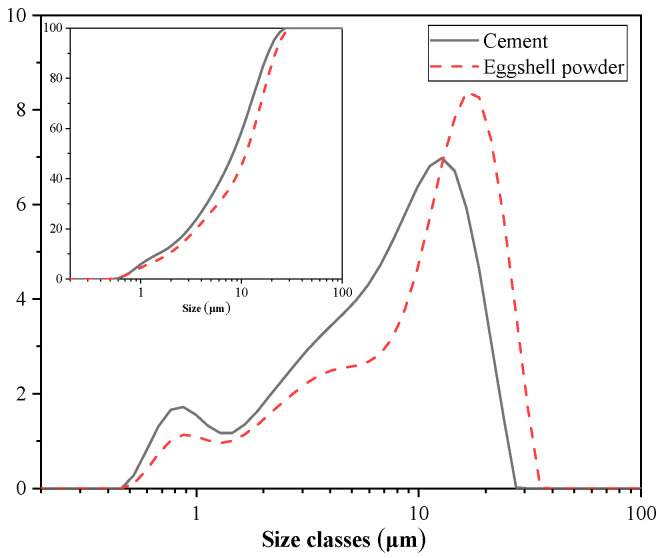
Particle size distributions of cement and eggshell powder.

**Figure 2:**
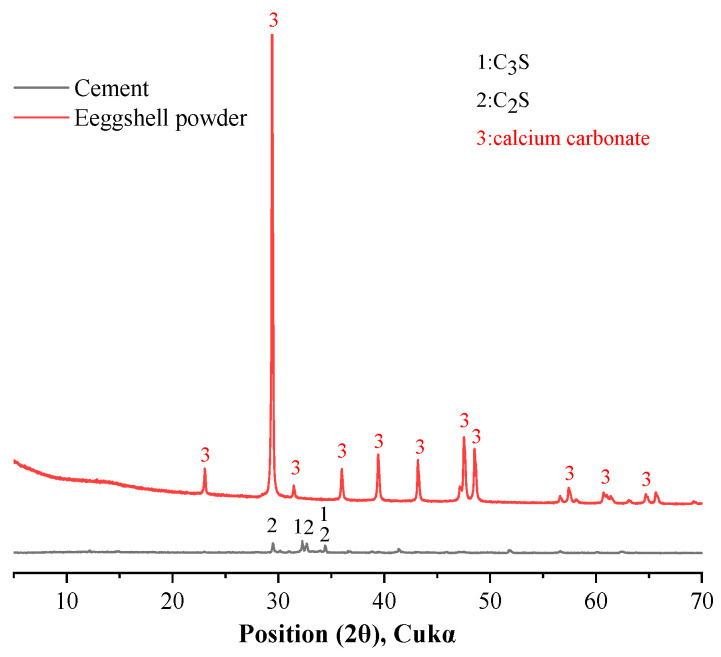
XRD patterns of cement and eggshell powder.

Binary mixtures were prepared from cement and eggshell powder. The cement type was Type I ordinary Portland cement, supplied by Sung Shin Cement Company (Seoul, Republic of Korea). The eggshells were obtained from a restaurant in Gangwon Province, South Korea. The washed eggshells were dried at 80 °C for 24 h and then ground with a ball mill to make eggshell powder. The particle size distribution and cumulative particle size distribution of cement and eggshell powder measured by a particle size distribution (Mastersizer 3000; Malvern Instruments Ltd., Almelo, The Netherlands) analyzer are shown in Figure 1. The average particle sizes of cement and eggshell powder were 8.23 μm and 10.92 μm, respectively. Table 1 shows the chemical composition of cement and eggshell powder measured by XRF (ZSX Primus II; Rigaku, Tokyo, Japan). It should be noted that the main component of eggshell powder is CaCO_3_, and the products of calcium carbonate decomposition are CaO and CO_2_, so the LOI_X_ of eggshell powder is relatively high. The densities of cement and eggshell powder were determined to be 3.14 g/cm^3^ and 2.89 g/cm^3^, respectively, according to the ASTM C188 [18] standard. The XRD patterns of cement and eggshell powder are shown in Figure 2. In the XRD pattern, the main crystalline phases of cement are C_2_S and C_3_S; the main crystalline phase of eggshell powder is calcium carbonate.

### 2.2. Mixing Ratio and Sample Preparation

Three sets of experimental samples were prepared, including pure paste samples and mortar samples. The water/binder ratio of all mixtures was 0.5 [19]. Table 2 lists the detailed mixing proportions of cement, eggshell powder, and water in all mixtures. In the mixture, cement was replaced by eggshell powder at 0%, 7.5%, and 15% substitution rates. The names representing the above mixtures are ES0, ES7.5, and ES15, respectively. This study only considered the single variable of eggshell powder. The ratio of sand to cementitious material was 2:1.

The weights of cement, eggshell powder, and water were measured in proportion and were kept indoors at a temperature of 20 °C. The paste was prepared using a mixer according to ASTM C305 [20] standards. The prepared slurry was used for microscopic performance testing. The sample was wrapped with film to prevent moisture loss, and then was placed in a 20 ± 2 °C curing box for solidification. After 1 day, the mold was removed. In the same way, mortar samples were prepared. All the paste and mortar specimens were sealed for curing using a wrap at a temperature of 20 ± 2 °C. The prepared mortar was poured into cuboid and cubic molds with dimensions of 40 × 40 × 160 mm and 50 × 50 × 50 mm. The prepared mortars were used for macroscopic performance testing.

### 2.3. Test Methods

#### 2.3.1. Heat of Hydration

Using an isothermal calorimeter (TA Instruments Delaware, New Castle, DL, USA), an isothermal calorimetry experiment at 20 °C was conducted on 5 g of the clean slurry to monitor the reaction heat in the first 7 days [21]. In order to minimize the interference of ambient heat on the samples, the indoor temperature was maintained at around 20 °C [22].

#### 2.3.2. Compressive Strength

Compressive strength tests were performed on mortar samples cured for 1, 3, 7, and 28 days according to ASTM C349 [23] standards. A set of three replicate tests was conducted to determine the average.

#### 2.3.3. Ultrasonic Pulse Velocity (UPV) and Surface Resistivity

UPV and surface resistivity tests were performed on samples sealed for 1, 3, 7, and 28 days [24]. The selected test piece was a 40 × 40 × 160 mm cubic mortar sample. Three samples were tested and their averages were calculated.

#### 2.3.4. X-ray Diffraction (XRD) and Fourier Transform Infrared Spectroscopy (FTIR)

XRD and FTIR analyses were performed on the clean pulp samples cured for 1 and 28 days. A sample was taken from the inside of the sample and then ground into a powder for testing. The operating current and voltage of XRD analysis were 30 mA and 40 kV, respectively. Scans were taken from 5° to 60° in 0.013° increments. FTIR analysis collected spectra in the range 4000–500 cm^−1^ with a resolution of 0.4 cm^−1^.

#### 2.3.5. Thermogravimetric Analysis (TGA)

TG was used to characterize the paste samples cured for 1 and 28 days. For about 20 mg of powder, the heating rate was 10 °C/min and the temperature range was 25~1000 °C. During the experiment, protective nitrogen gas was passed.

## 3. Results

### 3.1. Heat of Hydration

**Figure 3:**
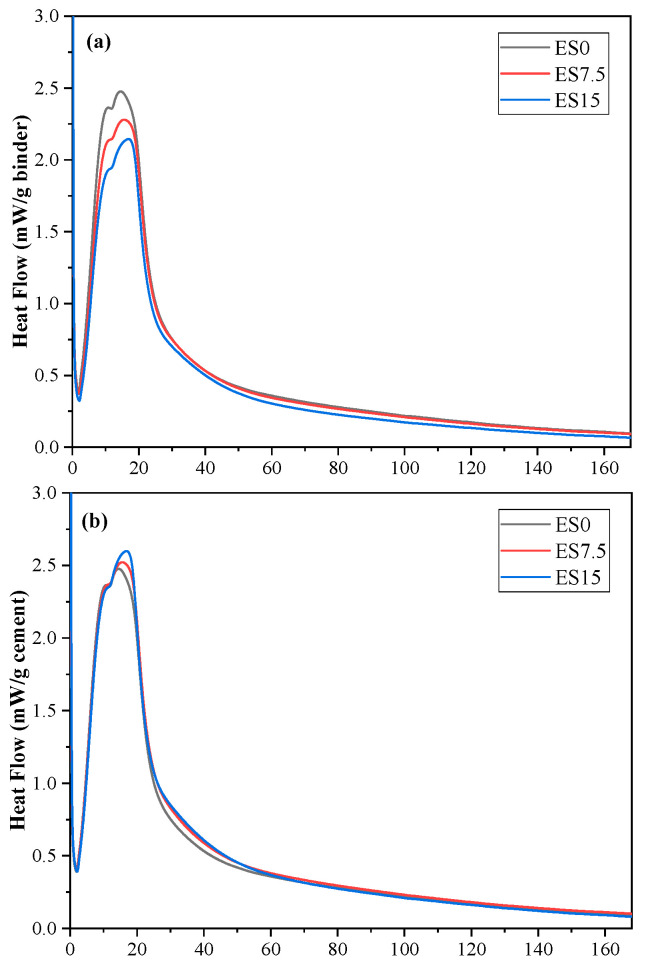

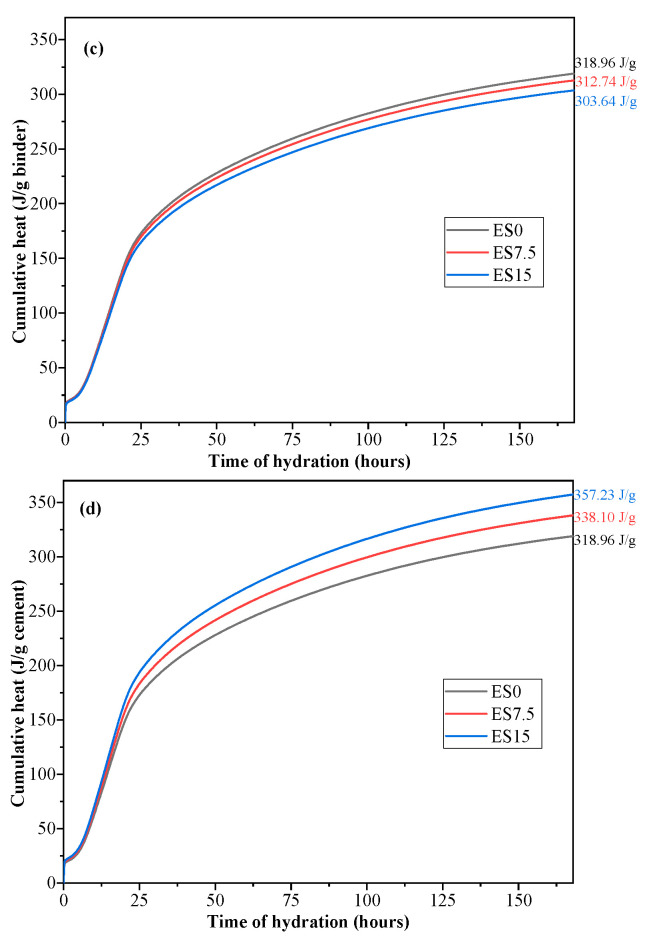
Hydration heat. (**a**) Heat flows of ES0, ES7.5, and ES15 (based on binder); (**b**) heat flows of ES0, ES7.5, and ES15 (based on cement); (**c**) cumulative hydration heat of ES0, ES7.5, and ES15 (based on binder); (**d**) cumulative hydration heat of ES0, ES7.5, and ES15 (based on cement).

The influence of eggshell powder on the cement hydration process includes the following two parts: condensation nucleation and dilution effects. Figure 3a,b show the hydration heat flow curves of the slurry samples cured for 168 h based on cementitious materials and cement, respectively.

As shown in Figure 3a, at the moment when the cement contacts water, heat is rapidly released, which is mainly attributed to the rapid reaction of C_3_A [25]. The main heat flow peak of cement hydration occurs at approximately 12.5 h. The appearance of the main peak is mainly due to the hydration reaction of C_3_S [25,26], and the intensity of the peak decreases as the eggshell powder content increases. This is attributed to the dilution effect caused by the addition of eggshell powder [27]. A higher peak appears between 17 and 23 h due to the secondary dissolution of C_3_A [26]. Studying the hydration heat flow curve based on cement in Figure 3b, it can be found that, as the eggshell powder content increases, the intensity of the peak increases slightly. This is due to the nucleating effect of the eggshell powder.

Figure 3c shows the cumulative heat of hydration based on each gram of gelling material, and it can be observed that, as the eggshell powder content increases, the cumulative heat of hydration decreases. This is mainly due to the dilution effect of the eggshell powder. In contrast, Figure 3d shows the cumulative heat of hydration on a per gram basis of cement. When calculating the cumulative heat of hydration release based on cement normalization, the cumulative heat of hydration of the ES7.5 and ES15 samples containing eggshell powder at 168 h is higher than that of the ES0 sample. After 168 h of hydration, the cumulative heat of hydration based on cement added with 0%, 7.5%, and 15% eggshell powder is 318.96, 338.10, and 357.23 J/g, respectively. The increase in the cumulative heat of hydration per gram of cement is attributed to the nucleation effect of eggshell powder. The presence of eggshell powder promotes the precipitation of hydration products. The precipitation of hydration products releases more accumulated hydration heat, thereby increasing cement hydration [28].

### 3.2. Strength

**Figure 4:**
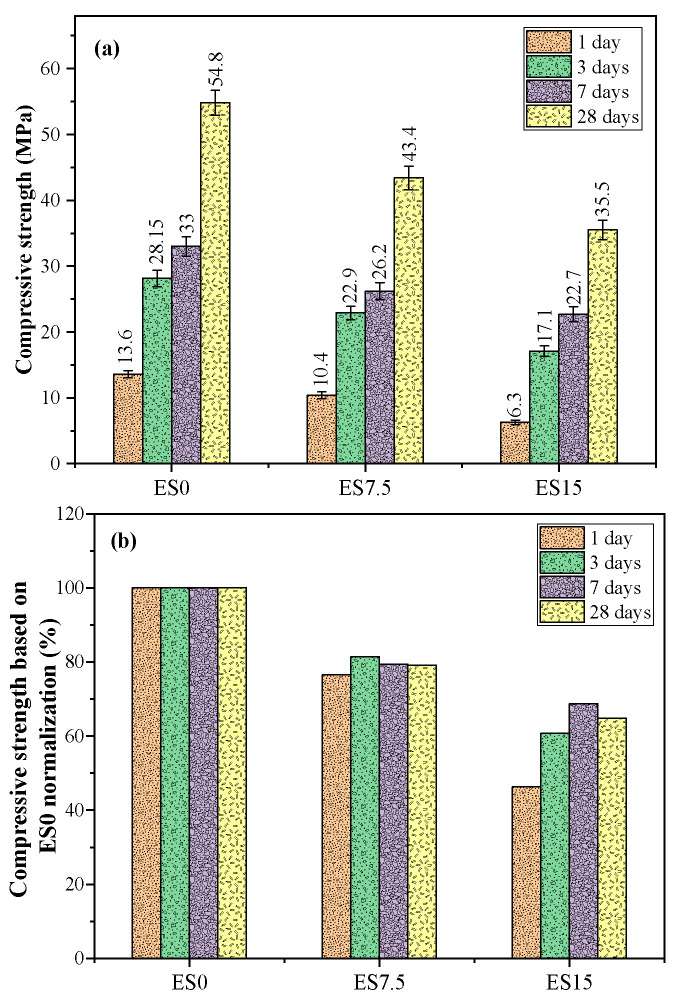
(**a**) Compressive strength results of ES0, ES7.5, and ES15 at different ages; (**b**) compressive strength based on ES0 normalization.

The addition of eggshell powder affects the hydration of cement and affects the final compressive strength. This study analyzes the changes in the compressive strength of mixture samples with different eggshell powder contents. Figure 4 shows the compressive strength trends of the mixture samples after curing for 1, 3, 7, and 28 d.

The compressive strength values of ES0, ES7.5, and ES15 mortar samples at 1, 3, 7, and 28 days are shown in Figure 4a. Figure 4b shows the compressive strength percentage results based on ES0 at each age. Compressive strength changes with eggshell powder content and age. It can be seen from Figure 4a that no matter how the content of eggshell powder changes, the compressive strength increases with the increase in curing age. It can also be seen from Figure 4a that the compressive strength of all mortar samples decreases as the eggshell powder content increases, regardless of age changes. This is because eggshell powder is mainly composed of calcium carbonate, whose lower reactivity increases porosity and reduces compressive strength. In addition, as the eggshell powder content increases, the cement content decreases, and the decrease in cement content will also reduce the compressive strength. The compressive strengths of ES0, ES7.5, and ES15 samples at 28 days of age are 54.8, 43.4, and 35.5 MPa, respectively. It can be seen that eggshell powder has a greater negative impact on compressive strength. There is a difference in the mechanics of strength and heat of hydration. Strength is closely related to the hardness of the material used. Compared with other hydration products, eggshell powder has low hardness, resulting in low strength. So, the reason for the decrease in strength is the molding process. On the other hand, the heat of hydration is mainly closely related to chemical reactions and has little to do with the hardness of the eggshell powder.

The data obtained by experiments always have certain differences. Even if repeated experiments or sampling are performed under the same conditions, the final data obtained are different. In this study, standard deviation is added to show the experimental data when processing the experimental data. The coefficients of variance of test results of strength are less than 5%.

### 3.3. Ultrasonic Pulse Speed

The performance of cement concrete can be evaluated using UPV test results [13,29]. The test results of UPV are affected by many factors, such as aggregate type, curing age, water/cement ratio, cement content, moisture content, porosity, etc. [30,31,32,33,34]. This study is based on cement and considers the impact of eggshell powder content on UPV test results.

**Figure 5:**
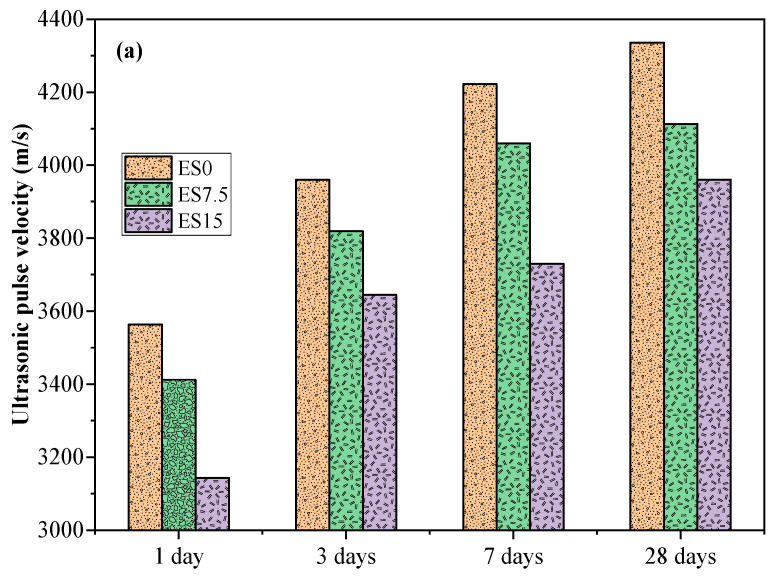

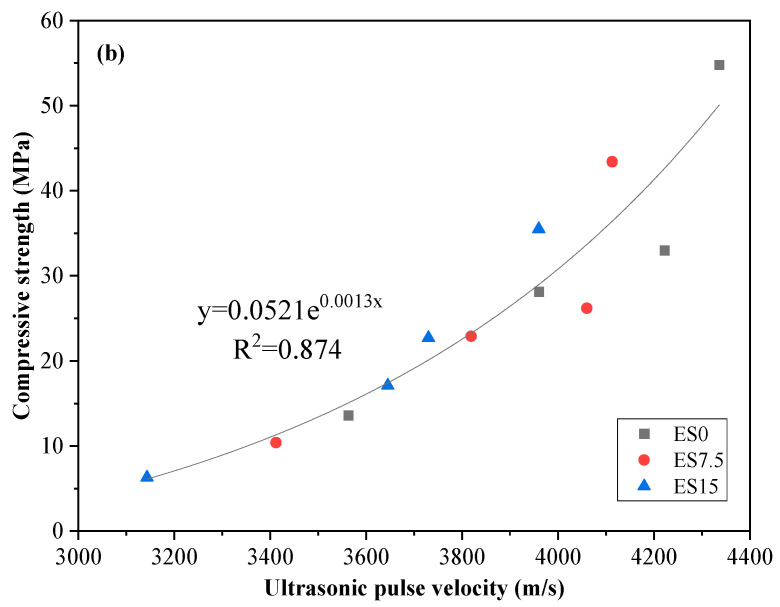
(**a**) Ultrasonic pulse velocity of ES0, ES7.5, and ES15; (**b**) relationship between UPV test results and compressive strength.

As shown in Figure 5a,b, the ultrasonic pulse speed gradually increases with the increase in curing age, and the pores are gradually filled (hydration occurs). The ultrasonic pulse speed of ES7.5 and ES15 mortar samples is lower than that of ES0. This is due to the reduced cement content in the mixture, which affects the pore space of the cement matrix [35]. Therefore, when the eggshell powder content increases, the ultrasonic pulse speed of the sample decreases. Several studies have shown [31,36,37] that ultrasonic pulse velocity can predict changes in compressive strength.

### 3.4. Resistivity

Previous studies have shown that the main factors affecting resistivity include saturation, pore distribution, conductive ion concentration, and the content of solid hydration products [38,39,40]. This study only considers the influence of a single variable—eggshell powder—on the resistivity of mortar samples.

**Figure 6:**
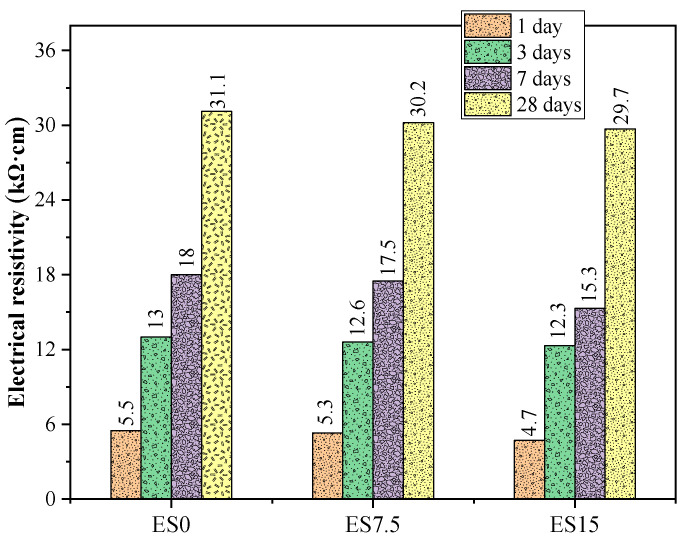
Resistivity test results of ES0, ES7.5, and ES15.

The resistivity test results of the mortar samples at 1, 3, 7, and 28 days of age are shown in Figure 6. The resistivities of ES0, ES7.5, and ES15 mortar samples at 1 day of age were 5.5, 5.3, and 4.7 kΩ·cm, respectively. The resistivities of ES0, ES7.5, and ES15 mortar samples at the age of 28 days were 31.1, 30.2, and 29.7 kΩ·cm, respectively. It can be found that as age increases, the resistivity of the mortar samples gradually increases. This is because cement hydration continues to decrease as age increases. As the eggshell powder content increases, the resistivity gradually decreases. Experiments show that eggshell powder can effectively reduce the resistivity of mortar samples. This is because eggshell powder is primarily composed of calcium carbonate, whose lower reactivity increases porosity and reduces resistivity.

### 3.5. XRD

**Figure 7:**
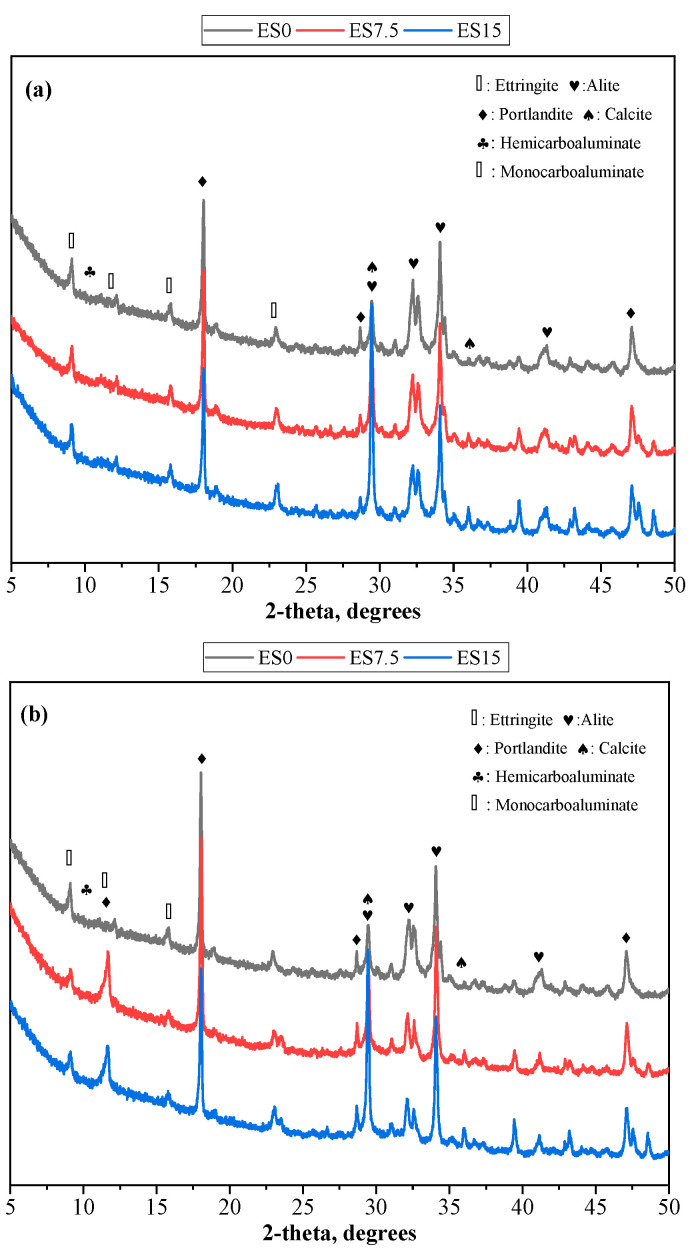
XRD patterns of ES0, ES7.5, and ES15. (**a**) 1 day; (**b**) 28 days.

The composition of the crystalline phase in the reaction product can be analyzed using XRD. Figure 7 shows the XRD spectra of ES0, ES7.5, and ES15 paste samples at 1 and 28 days of curing. Hydration products in the pure slurry samples include ettringite, portlandite, hemicarboaluminate, and monocarboaluminate [41]. In addition, there are unreacted alite and calcite crystal phases.

When studying Figure 7a,b, it can be found that as the eggshell powder content increases, the peak intensity of calcium carbonate increases. This is because eggshell powder is mainly composed of calcium carbonate. In addition, it can also be found that as age increases from 1 day to 28 days, the strength of portlandite becomes more obvious. This is because cement hydration continues to produce more portlandite. Hemicarboaluminate and monocarboaluminate are the reaction products of aluminate and calcium carbonate (mainly provided by eggshell powder; cement also contains a small amount of calcium carbonate) [42]. Comparing Figure 7a,b, it can be found that the peak of monocarboluminate at 1 day of age is not obvious, but the intensity of the peak at 28 days of age is enhanced. This is because hemicarboaluminate is first formed in the early stages of hydration and is gradually converted to monocarboaluminate with longer curing times [42,43]. As shown in Figure 7b, when the age reaches 28 days, the diffraction peak intensity of hemicarboaluminate for all samples is low, and the diffraction peak intensity of monocarboaluminate increases with the increase in eggshell powder content. As the eggshell powder content increases, the carbonate content increases, which is more conducive to the formation of monocarboluminate.

### 3.6. FTIR

**Figure 8:**
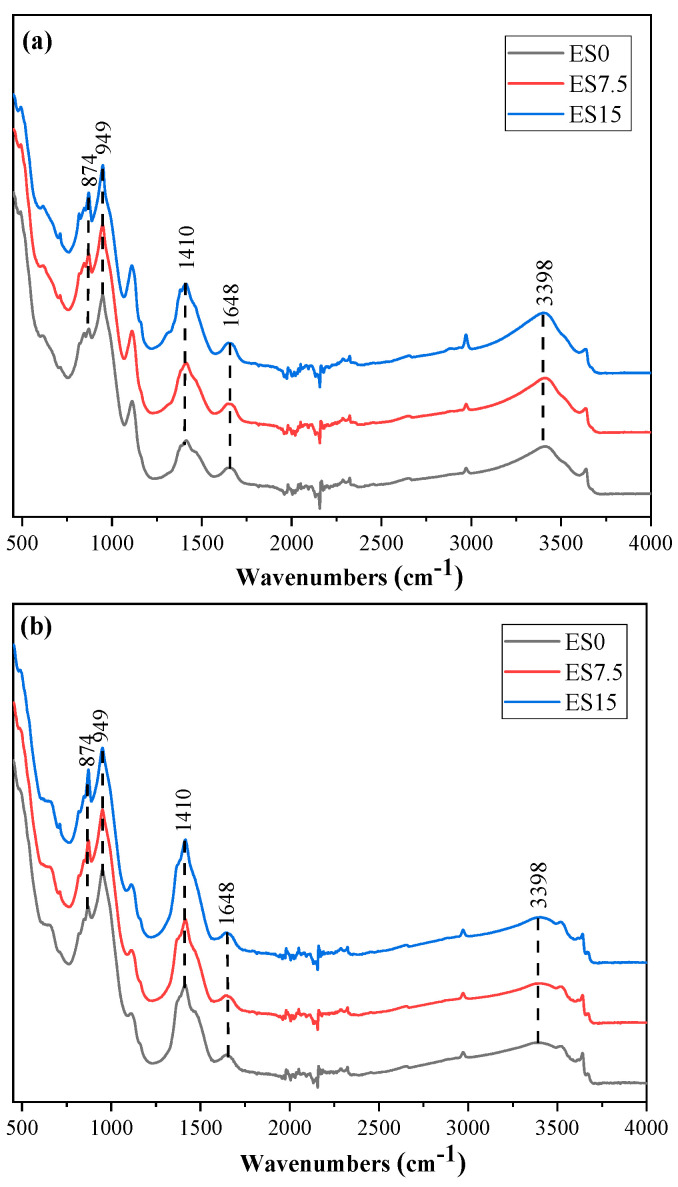
FTIR spectra of ES0, ES7.5, and ES15. (**a**) 1 day; (**b**) 28 days.

Conducting FTIR testing on hydration products can better understand the functional groups of hydration products. Figure 8 shows the FTIR spectra of ES0, ES7.5, and ES15 paste samples at 1 and 28 days. This study considers the effect of eggshell powder content on the mineral composition of hydration products. Table 3 lists the positions of the chemical bond wave numbers of the hydration products.

The absorption peak with a wave number of 3398 cm^−1^ is the stretching vibration of the O–H chemical bond, which corresponds to calcium hydroxide [44,45]. The bending vibration of the O–H bond appears in the wave number range of 1650–1640 cm^−1^ [44,45]. The peaks with wave numbers in the range of 997–938 cm^−1^ correspond to Si–O–Si (Al) asymmetric stretching vibration [47,48]. The hydration product corresponding to the Si–O–Si (Al) bond is CSH. As the amount of eggshell powder increases, the CSH content decreases slightly. The peak appearing at the wave number 1410 cm^−1^ is related to the asymmetric stretching vibration of the C–O bond. The reason why the intensity of the C–O bond absorption peak becomes more obvious is related to the content of eggshell powder.

### 3.7. TG-DTG

**Figure 9:**
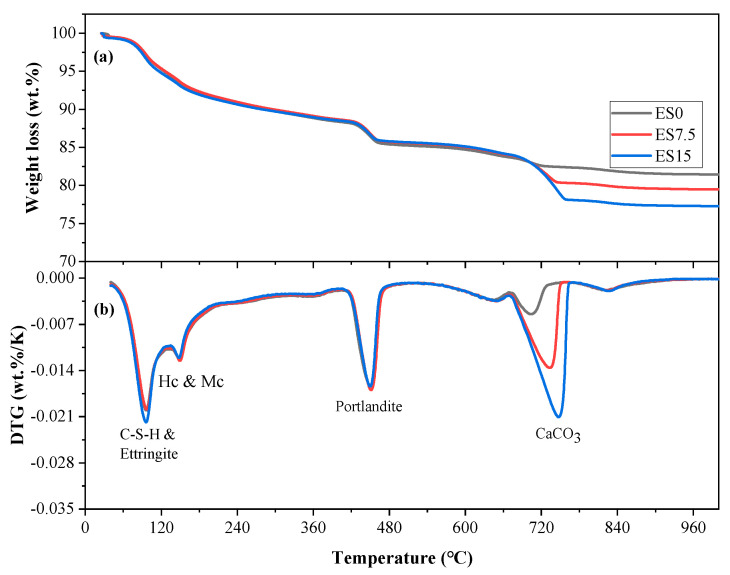
Curves of ES0, ES7.5, and ES15 for 1 day. (**a**) TGA; (**b**) DTG.

**Figure 10:**
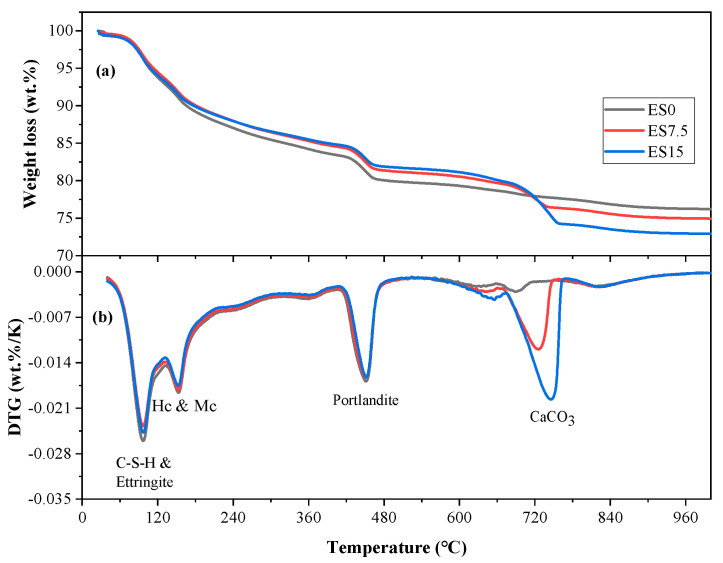
Curves of ES0, ES7.5, and ES15 for 28 days. (**a**) TGA; (**b**) DTG.

Figure 9 and Figure 10 show the TG-DTG curves of ES0, ES7.5, and ES15 paste samples at 1 and 28 days of age. The absorption peaks of the reaction products appear at around 100, 160, 450, and 750 °C, which are related to the decomposition of C–S–H and ettringite, hemicarboaluminate and monocarboaluminate, portlandite, and CaCO_3_, respectively [49].

When comparing Figure 9 and Figure 10, it can be found that from day 1 to 28 days, the peaks of C–S–H and ettringite are significantly enhanced. As age increases from day 1 to 28 days, cement hydration continues, producing more C–S–H and ettringite. In addition, it is also found that the decomposition peak of calcium carbonate is weakened. This is because the aluminate in the cement reacts with calcium carbonate to form hemicarboaluminate and monocarboaluminate, which consumes calcium carbonate. In addition, careful study finds that the control group ES0 also contains calcium carbonate. This is because the cement used in this study is mixed with a small amount of calcium carbonate [50].

Chemically bound water content and calcium hydroxide content are important parameters for studying cement hydration. We calculate the chemically bound water content of ES0, ES7.5, and ES15 clean slurry samples cured for 1 day and 28 days. The calculation according to Formula (1) [51] is as follows:

(1) $$H_{a}=\frac{M_{50}-M_{550}}{M_{550}}\times 100\%$$

where $M_{50}$ and $M_{550}$ are the masses of samples at 50 and 550 °C, respectively, and $H_{a}$ is the percentage of chemically bound water.

The calculation results of TGA are shown in Table 4. According to the results in Table 4, it can be observed that as the curing age increases from 1 day to 28 days, a significant increase in chemically bound water occurs. This is attributed to the continued hydration of the cement. In addition, regardless of the age change, as the eggshell powder content increases, the amount of chemically bound water decreases. The reduction in chemically bound water content is related to the following factors: (1) the nucleation effect of eggshell powder promotes hydration of cement and thereby increases the bound water content; (2) the reduction in cement content leads to a reduction in hydration products, thereby reducing the amount of chemically bound water. The bound water content depends on increasing factors competing with decreasing factors.

We performed quantitative calculations on the hydration product portlandite of ES0, ES7.5, and ES15 paste samples cured for 1 and 28 days. The calculation according to Formula (2) is as follows:

(2) $$F_{a}=\frac{M_{400}-M_{500}}{M_{500}}\times 100\%$$

where $M_{400}$ and $M_{500}$ are the masses of samples at 400 and 500 °C, respectively, and $F_{a}$ is the percentage of portlandite.

The calculation results of TGA are shown in Table 4. According to the results in Table 4, it can be observed that, as the curing age increases from 1 day to 28 days, portlandite generation increases significantly. This is attributed to the continued hydration of the cement. In addition, regardless of the age change, as the eggshell powder content increases, the portlandite content decreases. The reason for the decrease in portlandite production is consistent with the decrease in chemically bound water content. Although the additional nucleation effect of eggshell powder promotes the hydration of cement to produce more portlandite, it cannot be equivalent to the reduction in portlandite production caused by the reduction of cement. In addition, we also find that the calculated results of portlandite production are consistent with the XRD experimental results, that is, as the eggshell powder content decreases, the production of portlandite decreases. 

## 4. Hydration Model for Cement–Eggshell Powder Binary Blends

As shown in the previous experimental section, for eggshell powder, it mainly plays the dilution effect, nucleation effect, and chemical reaction effect. For specimens with a water/binder ratio of 0.5, the dilution effect has a limited effect. In addition, compared with other cementitious materials, such as slag and fly ash, the chemical reaction effect of eggshell powder is not obvious. Therefore, in our simulations, the nucleation effect of eggshell powder is mainly considered. This simulation consists of two parts. The first part is to determine the model parameters. The second part is to use the model to extrapolate the results of the hydration heat and estimate the strength.

### 4.1. Calibration of Parameters of the Hydration Model

The three-parameter equation (TPE) for hydration heat is shown as follows [52]:

Q(t) = Q0 × exp(−b × (t^c))(3)

where Q(t) is the test results of hydration heat, Q0 is the hydration heat of 1 g fully hydrated cement, b is the reaction rate parameter, and c is the shape parameter.

Based on the test results of the hydration heat normalized by cement mass (shown in Figure 3d), the parameters of the hydration model are determined as shown in Table 5.

**Figure 11:**
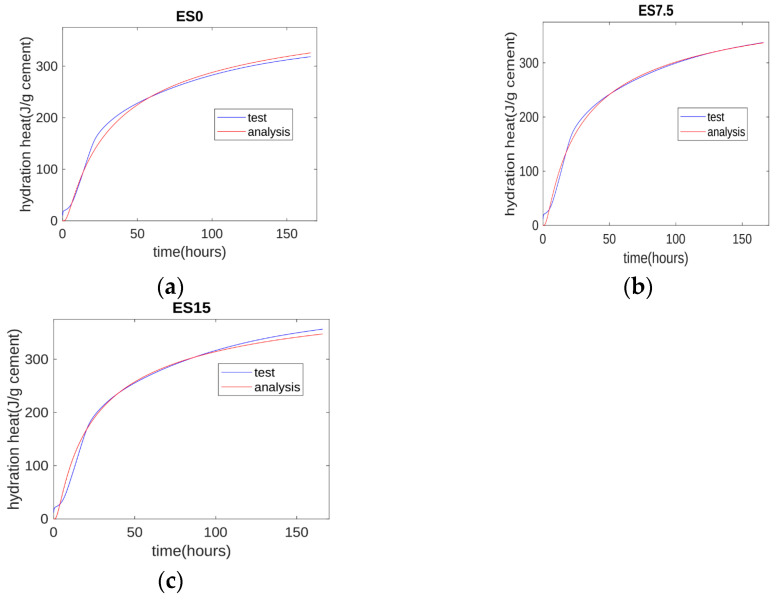
Hydration heat analysis. (**a**) Analysis results of control specimen; (**b**) analysis results of 7.5% eggshell specimen; (**c**) analysis results of 15% eggshell specimen.

The comparison between test results and analysis results is shown in Figure 11. The analysis results generally agree with the test results. At the early age of about 10 h, the test results are slightly higher than the analysis results. This is because the three-parameter equation model does not consider the hydration heat from the initial contact period.

### 4.2. Evaluation of Strength Development Using the Hydration Model

**Figure 12:**
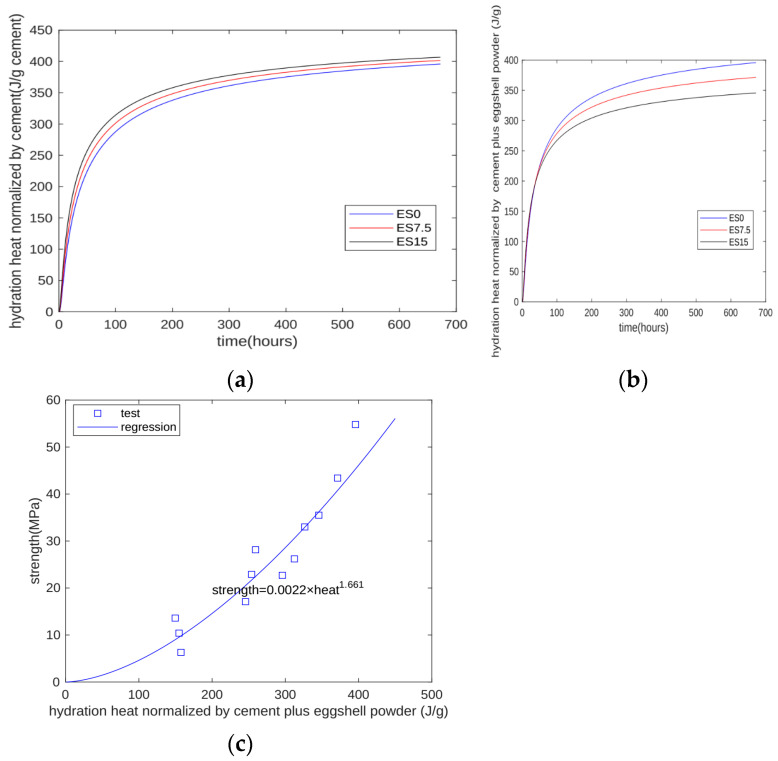
Prediction of strength development using proposed model. (**a**) Heat normalized by cement mass; (**b**) heat normalized by cement plus eggshell powder mass; (**c**) relation between strength and hydration heat.

In cement–eggshell blends, eggshell is almost a chemical inert filler. For the hydration heat test, due to limited test equipment, only the first 7 days of heat can be measured. However, based on the proposed model, we can calculate the hydration heat beyond 7 days. The calculated results are shown in Figure 12. Figure 12a shows the heat normalized by cement mass. As the eggshell powder content increases (due to the nucleation effect), the heat normalized by cement mass increases. Figure 12b shows the heat normalized by cement plus eggshell powder mass. As the eggshell powder content increases (due to the dilution effect), the heat normalized by cement plus eggshell powder mass decreases. This means that, although the nucleation effect can accelerate cement hydration, it cannot compensate the reduction in the dilution effect. 

Figure 12c shows the relation between strength and calculated hydration heat. Generally, for all three specimens at all test ages of 1, 3, 7, and 28 days, strength is an exponential function of hydration heat. The coefficient of determination of regression is 0.9045. In summary, our proposed model can overcome the weak point of the test machine of hydration heat and the extrusion of the test results of hydration heat to a longer age.

## 5. Discussions and Conclusions

### 5.1. Discussions

At present, compared with supplementary cementitious materials and filler materials, research regarding the use of eggshell powder to replace cement is relatively limited, so we conducted a series of macro- and micro-experimental studies, and presented a hydration model to clarify the macro- and micro-mechanisms of eggshell powder on cement-based materials.

In this study, the substitution rates of eggshell powder are 7.5% and 15%. The chemical composition of eggshell powder is similar to limestone powder, which is calcium carbonate. Compared with other filler materials, such as limestone powder (generally 5–20%), the replacement rate of eggshell powder in this study is also within the normal range. 

Some researchers report that when 5% of cement is replaced by limestone powder, the change in compressive strength is not significant. Our results show that when 7.5% cement is replaced by eggshell powder, the compressive strength is reduced, and the degree of reduction cannot be ignored. The authors speculate that this difference may come from the difference in the hardness of eggshell powder and limestone powder rather than from the substitution amount. In other words, we admit that the results we obtained are relatively negative experimental results; however, these results also have implications for the engineering community, that is, caution is needed when choosing eggshell powder to replace cement.

The eggshell powder in this study is used directly. Of course, the performance of eggshell powder concrete may be improved after appropriate physical or chemical treatment. This needs to be discussed in our future research.

The authors believe that the most important originality of this article is the proposed hydration model, through which the development of compressive strength and hydration heat of cement-based materials containing eggshell powder is predicted. According to the authors’ review of the literature, current research on eggshell powder concrete is basically experimental research, and research on the eggshell powder hydration model has not yet been carried out. This paper proposes a hydration model for eggshell powder concrete for the first time. The development of hydration heat and compressive strength can be predicted through the proposed hydration model. The prediction of the heat of hydration can be used for the temperature development evaluation of mass concrete, and the development of compressive strength can be used for the evaluation of formwork removal time of the structure during construction. Additionally, on the basis of compressive strength, structural designers can design relevant structural elements or evaluate other properties of the concrete, such as elastic modulus and tensile strength development.

### 5.2. Conclusions

This article conducted a series of studies on the macro- and micro-properties of cement-based materials mixed with eggshell powder, and the main conclusions are as follows:

Experiments on the heat of hydration found that, as the eggshell powder content increases, the cumulative heat of hydration decreases. This is mainly due to the dilution effect of eggshell powder. The increase in the cumulative heat of hydration per gram of cement is attributed to the nucleation effect of eggshell powder. The presence of eggshell powder promotes the precipitation of hydration products. The precipitation of hydration products releases more accumulated hydration heat, thereby increasing the hydration of cement. The compressive strengths of ES0, ES7.5, and ES15 samples at 28 days of age are 54.8, 43.4, and 35.5 MPa, respectively. It can be seen that eggshell powder has a greater negative impact on compressive strength. When the eggshell powder content increases, the ultrasonic pulse speed of the sample decreases. The effect of eggshell powder on the speed and intensity of ultrasonic waves has a similar trend. As the eggshell powder content increases, the resistivity gradually decreases. Experiments show that eggshell powder can reduce the resistivity of mortar samples. This is because eggshell powder is primarily composed of calcium carbonate, whose lower reactivity increases porosity and reduces resistivity.

The XRD experiment found that the peak of monocarboluminate is not obvious at 1 day of age, but the intensity of the peak increases at 28 days of age. This is because hemicarboaluminate first forms in the early stages of hydration and gradually converts to monocarboaluminate over longer curing times. Regarding the FTIR experimental results, the peaks with wave numbers in the range of 997–938 cm^−1^ correspond to the asymmetric stretching vibration of Si–O–Si (Al), and the corresponding hydration product is CSH. As the amount of eggshell powder increases, the CSH content decreases slightly. TG’s experiment found that the aluminate in cement reacts with calcium carbonate to produce hemicarboaluminate and monocarboaluminate, which consume calcium carbonate. As the eggshell powder content increases, the portlandite content decreases. Although the additional nucleation effect of eggshell powder promotes the hydration of cement and produces more portlandite, it cannot offset the reduction in portlandite production caused by the reduction of cement.

A numerical hydration model is proposed for cement–eggshell powder binary blends. The parameters of the hydration model are determined based on the hydration heat normalized by cement mass. Moreover, hydration heat until 28 days is calculated using the proposed model. The strength development of all specimens and all test ages can be expressed as an exponential function of hydration heat. The coefficient of determination of regression is 0.9045.

The research in this article indicates that excessive use of eggshell powder in cement can reduce the mechanical properties of cement concrete. In engineering practice, eggshell powder needs to be used with caution to replace cement.

## Figures and Tables

**Table 1:** Chemical compositions of cement and eggshell powder.

Identification	SiO_2_	Al_2_O_3_	CaO	Fe_2_O_3_	MgO	Na_2_O	K_2_O	ZnO	TiO_2_	SO_3_	LOI
Cement	19.40	4.46	64.20	3.00	2.29	0.12	0.98	0.06	0.23	3.83	1.43
Eggshell powder	0.02	0.02	74.2	-	0.69	0.14	0.07	-	-	0.34	24.52

**Table 2:** Mixtures of samples.

	Group	OPC	Eggshell Powder	Sand	Water	Water/Binder
Paste	ES0	100	0	-	50	0.5
	ES7.5	92.5	7.5	-	50	0.5
	ES15	85	15	-	50	0.5
Mortar	ES0	100	0	200	50	0.5
	ES7.5	92.5	7.5	200	50	0.5
	ES15	85	15	200	50	0.5

**Table 3:** Positions of infrared bands and functional groups in FTIR spectra.

Wavenumber (cm^−1^)	Functional Groups	Reference
3457–3390	H–O–H, υ	[44,45]
1650–1640	H–O–H, δ	[44,45]
1400–1500	C–O, υ_as_	[46]
997–938	Si–O–Si, υ_as_	[47,48]
853–600	Al–O–H, δ	[47]

**Table 4:** Combined water, portlandite, and calcium carbonate of ES0, ES7.5, and ES15 at 1 day and 28 days.

wt%	ES0	ES7.5	ES15
Combined water (1 day)	13.15	12.76	11.99
Portlandite (1 day)	14.80	14.59	13.57
Combined water (28 days)	19.55	18.29	16.91
Portlandite (28 days)	18.48	17.18	15.68

**Table 5:** Parameters of hydration model.

Q0	b0	b7.5	b15	c
456.04	7.96	7.16	6.44	−0.62

b0, b7.5, and b15 are reaction rate parameter b for control, 7.5% eggshell, and 15% eggshell specimens, respectively. For these three specimens, the values of Q0 and c are the same, and only the value of b is different. This means that eggshell can accelerate the hydration of cement but does not change the essence of cement hydration.

## Data Availability

The data presented in this study are available from the corresponding author upon reasonable request.
